# Sex-Specific Transcriptome Differences in Human Adipose Mesenchymal Stem Cells

**DOI:** 10.3390/genes11080909

**Published:** 2020-08-08

**Authors:** Eva Bianconi, Raffaella Casadei, Flavia Frabetti, Carlo Ventura, Federica Facchin, Silvia Canaider

**Affiliations:** 1 National Laboratory of Molecular Biology and Stem Cell Bioengineering of the National Institute of Biostructures and Biosystems (NIBB)—Eldor Lab, at the Innovation Accelerator, CNR, Via Piero Gobetti 101, 40129 Bologna, Italy; eva.bianconi2@gmail.com (E.B.); carlo.ventura@unibo.it (C.V.); silvia.canaider@unibo.it (S.C.); 2 Department for Life Quality Studies (QuVi), University of Bologna, Corso D’Augusto 237, 47921 Rimini, Italy; r.casadei@unibo.it; 3 Department of Experimental, Diagnostic and Specialty Medicine (DIMES), University of Bologna, Via Massarenti 9, 40138 Bologna, Italy; flavia.frabetti@unibo.it

**Keywords:** sex, sex dimorphism, mesenchymal stem cells, human adipose derived stem cells, microarray, gene expression

## Abstract

In humans, sexual dimorphism can manifest in many ways and it is widely studied in several knowledge fields. It is increasing the evidence that also cells differ according to sex, a correlation still little studied and poorly considered when cells are used in scientific research. Specifically, our interest is on the sex-related dimorphism on the human mesenchymal stem cells (hMSCs) transcriptome. A systematic meta-analysis of hMSC microarrays was performed by using the Transcriptome Mapper (TRAM) software. This bioinformatic tool was used to integrate and normalize datasets from multiple sources and allowed us to highlight chromosomal segments and genes differently expressed in hMSCs derived from adipose tissue (hADSCs) of male and female donors. Chromosomal segments and differentially expressed genes in male and female hADSCs resulted to be related to several processes as inflammation, adipogenic and neurogenic differentiation and cell communication. Obtained results lead us to hypothesize that the donor sex of hADSCs is a variable influencing a wide range of stem cell biologic processes. We believe that it should be considered in biologic research and stem cell therapy.

## 1. Introduction

Mammalian sexes can manifest their differences in many ways, leading to the so-called “sexual dimorphism” (SD), an evolutive strategy for individuals to successfully mate. Male and female have divergent sex-specific traits that allow a fast recognition of the putative sexual partner, giving benefits both at the single individual and at the species level [1]. In humans, the SD is studied in-depth in a broad range of knowledge fields and the differences are related in part to the circulating hormones, but they do not provide the entire explanation for these sex differences. For instance, anthropometry, neurosciences, psychiatry, metabolism and immunology offer a huge number of SD evidence. The female and male hip bone are structurally different as adaptation to birth [2] and this difference could be successfully used for the sex determination [3,4]. Different patterns of functional lateralization between men and women are reported [5]. In the last decades, the processing of verbal and spatial information was well documented as sex-related, with the female brain more symmetrically organized than the male one [6,7,8,9,10]. However, recently, a critical review of literature suggests that cognitive and hemispheric asymmetry sex differences are at least partly independent of each other [11]. Sex-biased differences, not only in the brain, influence the predisposition to some neurological and psychiatric disorders and lead to related phenotypes [12,13,14]. For example, autism spectrum disorder (ASD) incidence shows a male-to-female ratio of four to one, while that of attention deficit hyperactive disorder (ADHS) is 10-fold higher in boys [13,15] and several addictive’s abuses are documented as sex-biased and reviewed in [14]. Moreover, fatty acid metabolism and kinetics are different both at basal levels and during exercise, with a higher rate of plasmatic appearance of glycerol [16] and of fatty acids in women than in men [17]. Lastly, men and women show different immune system already from intrauterine life with a different susceptibility to pathogens [18]. Male fetuses experience a more inflammatory uterine environment [19] and have higher level of IgE than the female ones [20], while, in postnatal life, males have a more severe response to sepsis [21] and are more susceptible to some bacterial and viral infections [22,23]. Moreover, it is increasingly evident that also cells differ according to sex, irrespective of their history of exposure to sex hormones. Researchers have found, for example, that human cells isolated from male or female donors have different concentrations of metabolites [24] or that cells derived from male and female mice have a different response to stress [25].

The karyotype is the first great difference between sexes, and it is at the basis of all the sexually dimorphic traits. The presence or the absence of the Y chromosome allows to determine the gonadal sex of an individual and this implies that, excluding Y chromosome genes, males and females have the same genome [26]. Despite this, sex-related differences arise at the molecular level due to differential gene expression [27,28], differential alternative transcripts [29,30] and/or epigenetic modifications [31]. Transcriptional differences are a consequence of the genetic sex and are not strictly dependent on the sexual hormones. In fact, in pre-implanted embryos and before the gonadal formation, there is already a sex-biased gene expression in several species, including humans [32]. Only in male embryos, Y-linked genes are transcribed [33,34]. At the same time, after the embryonic genome activation and up to the process of X-inactivation, both X chromosomes are active with a consequent higher expression of X-linked genes in female embryos [35,36]. These different gene expressions could affect the variation of autosomal genes. For instance, the expression of the Y-linked *SRY* gene activates a specific set of genes for testes formation, as *SOX9* and *FGF9* both in mice and humans, and, at the same time, represses female specific genes as *WNT4* [37]. At least in mouse, the major molecular differences between sexes in gene expression are in gonadal tissues [26,38], but diversity occurs also in the other organs as recently reported by Gershoni and Pietrokovski [39] and are well documented in liver [40], brain [41,42] and heart [43]. Moreover, according to a recent study, some of the imprinted genes closely associated with the control of fetal growth rates and expressed in the hypothalamus, an important target for gonadal hormones, seem to be controlled or at least affected, by sexual differentiation and interestingly exhibit different sexual expression [44].

In the context of SD that manifests itself at different levels of the living beings, our interest falls at the cellular level, still little studied and poorly considered when cells are used in scientific research [45].

Specifically, we have studied, although still scarce, the scientific literature on SD at the level of mesenchymal stem cells (MSCs), our main object of study. Sex differences in MSCs are described in animal and human cells, with particular regard to the differentiation process and cellular functions. In murine models, osteoblastogenesis is sexually dimorphic and influenced by genetic factors, with a higher expression of *Rankl* and *Opg* in female osteoblasts [46], as well as it is reported a delayed bone healing in female rats associated with a diminished number of MSCs [47]. In rhesus monkeys, the neurogenic potential is different between female and male MSCs. In fact, nestin-positive female MSCs show a higher neurogenic potential accompanied by increased synthesis and excretion of GABA, compared with the male counterparts [48]. A different paracrine MSC function was indicated as sex-dependent; for instance, rat female MSCs produce less proinflammatory cytokines and more growth factors than male MSCs [49]. In particular, it was shown that the higher production of growth factors in female MSCs led to a greater recovery of left ventricular developed pressure when MSCs are infused in infarcted rat hearts [50]. A different production of cytokines is also reported in piglets, with a higher production of IL-6 by male MSCs; at the same time, MSCs derived from adipose tissue of young female pigs were more resistant to senescence in vitro [51]. Muscle-derived stem cells transplanted into dystrophic mice regenerated skeletal muscle more efficiently when derived from female donors [52]. Even in human stem cells, sex differences are described. For instance, during cardiac differentiation of human embryonic stem cells (hESCs) there is a differential expression of the male-specific region of the Y chromosome genes and of their X chromosome counterparts [53]. A different transcriptomic profile was detected in the trophoblastic progenitors and also during the differentiation process itself [54]. However, regarding adult MSCs, literature is not abundant; Aksu et coll. [55] reported that the human adipose-derived stem cells (hADSCs) isolated from males were more osteogenic than those from females and, at the same time, male MSCs derived from the Wharton’s jelly (hWJ-MSCs) have a stronger expression of *OCT4,* a pluripotent stem cell marker and DNA–methyltransferase 1, respectively [56]. Recently, Serpooshan and coll. [57] have investigated nanoparticles uptake and reprogramming capacity of human amniotic stem cells (hAMSCs) of diverse sex. Female cells showed a greater uptake than male MSCs, with cell reprogramming efficiency being affected by hAMSC sex. In the same study, the different uptake was correlated to modifications of physical–chemical properties that affect nanoparticles–cell interaction due to the significant variations in the production of paracrine factors among male and female cells [57].

Deepening knowledge on stem cell biology and SD could be a useful and interesting tool to improve MSC applications in regenerative medicine. In fact, these cells represent a potential and important cell source: MSCs virtually reside in all adult organs, like adipose tissue, bone marrow and dental pulp, they are multipotent, relatively easy to expand, possessing anti-inflammatory, immunomodulatory and pro-angiogenic effects [58]. MSCs are also immune evasive [59]. Despite this, there is still poor knowledge of sex influence on MSC differentiation, proliferation, migration and senescence, as well as of cell sex-effects as a part of cell therapy. MSCs are studied and used as therapeutic mediators in multiple degenerative diseases and tissue injuries [60,61,62,63], but their application in cell therapies still requires remarkable optimization.

The aim of this study is to identify the transcriptomic differences between hADSCs derived from male and female donors. Fat is a discarded tissue after cosmetic surgery, but at the same time it is a rich source for stromal MSCs [64]. We performed a systematic meta-analysis of hADSC microarrays data using the Transcriptome Mapper (TRAM) software [65]. In the current study, this bioinformatic tool was useful to integrate and normalize datasets from multiple sources and allowed us to highlight which chromosomal segments and genes are differentially expressed in male and female hADSCs.

## 2. Materials and Methods

### 2.1. Database Search and Selection

The search for gene expression data related to hADSCs isolated from male and female donors was performed using the genomic repositories gene expression omnibus (GEO) [66] up to July 2019. The search parameters were: “adipose stem cells” and “array”, then filtered for “Homo sapiens” (organism) and “profiling expression by assay”. We selected those series including microarray experiments conducted on hADSCs isolated from subcutaneous fat of non-obese adult (>18 years old) [67] subjects (BMI < 30 kg/m^2^) [68,69] in which the donor sex was specified. Moreover, in selected arrays, hADSCs were cultured in standard conditions (DMEM, fetal serum and antibiotics) or enriched with fibroblast growth factor (FGF) as a supplement and they were analyzed in the short-term culture (i.e., subculture passage ranging from 1st to 4th). hADSCs submitted to pharmacological or other treatments were excluded by analysis.

Some GEO series were not included in TRAM analysis, as previously described [42], for the following criteria of exclusion: data from exon array or other probes (an extremely high number of data rows could interfere with program execution); the absence of identifiers corresponding to those found in the GEO sample records (GSM); platforms with an atypical number of genes (i.e., <5000 or >60,000); data with expression values not explicitly stated as linear or logarithmic.

### 2.2. Literature Search

A systematic literature search was conducted in order to identify additional articles related to global gene expression profile experiments in hADSCs and not reported in GEO. We start with a general search conducted on PubMed up to July 2019, using the terms “human mesenchymal stem cells” and “microarray analysis” and “human”. Then, a more advanced search was performed by using the medical subject headings (MeSH) terms “human mesenchymal stem cells”, “microarray analysis” (or “gene expression profiling” or “oligonucleotide array sequence analysis”) and “human”. Both searches produced no additional data. Another systematic literature search on PubMed was conducted in order to analyze bibliography about genes resulted differently expressed between sexes after TRAM analyses, using the terms “stem cells”, “sex” and “gender” in association with the gene name. This type of search has been made in addition to gene database (available at https://www.ncbi.nlm.nih.gov/gene) and Gene Cards database (available at https://www.genecards.org/) searches.

### 2.3. Tram Analysis

TRAM software is freely available at http://apollo11.isto.unibo.it/software. We used the pre-loaded TRAM version 1.3_HUMAN_2017 (December 2017), supplied for *H. sapiens* and replacing any previous version of TRAM_HUMAN [65,70].

At first, we checked for the presence in the TRAM 1.3 version of all platforms used in the array experiments. This is a necessary step, as during the import phase the software can associate a specific gene symbol via UniGene parsing with each probe identifier. Any additional platforms can be manually extracted and imported.

The following step of the setup phase was to download all the samples selected for each series, as tab-delimited text format. Samples were then divided into pools, according to the sex: hADSCs from male subjects (pool A) and hADSCs from female subjects (pool B). Female subjects were then also analyzed considering the culture method: hADSCs cultured in FGF-supplemented medium (pool C) and hADSCs cultured in a standard medium without FGF (pool D).

As previously stated, during the data import step the software is able to assign the appropriate gene symbol to each probe identifier through UniGene parsing and subsequently normalize them (via intra- and inter-sample normalization). This procedure makes it possible to compare gene expression data from experiments performed with different platforms and/or biologic conditions. Moreover, TRAM software can avoid the risk of bias as it is intrinsically resistant to the methodical differences between batches (groups) of samples [65].

TRAM analysis was performed both for pool A vs. B (named “TRAM sex”) and for pool C vs. D (named “TRAM FGF”) according to a standard setting for creation of transcriptome maps (“Map” mode), using both default and single gene level parameters [65,71]. Briefly, the “Map” mode analysis was first set to evaluate segments of 500,000 base pairs (bp) with a sliding window of 250,000 bp (default Map mode analysis). In these conditions, the expression value for each genomic segment is calculated as the mean of the expression values of all the loci included in that segment, defining it as over/underexpressed in a statistically significative manner if the expression value was different between the two conditions and contained at least three over/underexpressed genes (genes at the top/bottom 2.5% of values).

Afterwards, a second analysis was conducted for both “TRAM sex” and “TRAM FGF” with different parameters (single gene level Map mode analysis): the window size was set to 12,500 bp with a shift of 6250 bp. In this way the significant over/underexpression of a segment corresponds in most cases to that of a single gene [65].

In each type of analyses (default and single gene level) the new parameter “Sample Number”, available from TRAM 1.3 version, was set to the suggested value of *n* = 2, allowing the inclusion in the results of genes having at least “n” values across the analyzed datasets (when dataset number is greater than 1). The statistical significance was calculated taking into account all genes in the genome (genome median) and corrected for multiple comparisons possible causing false discovery rate (FDR) due to the high number of segments or genes in a genome (*q*-value). A segment or a gene was considered to be statistically significantly over- or underexpressed for *q* < 0.05 [65].

The 20 most over- and underexpressed genes, as resulted from single gene level analysis, were compared to point out the intersection between “TRAM sex” and “TRAM FGF”.

### 2.4. Other Analyses

UniGene [72], National Center for Biotechnology Information (NCBI) Entrez Gene [73] and Gene ontology (GO) [74] were used to obtain gene-specific information and to functionally characterize the set of genes derived from TRAM analyses. Moreover, the ShinyGo web application tool [75] was employed for enrichment analysis using the gene list resulted from TRAM analysis at single gene level.

## 3. Results

### 3.1. Database and Literature Search

**Figure 1:**
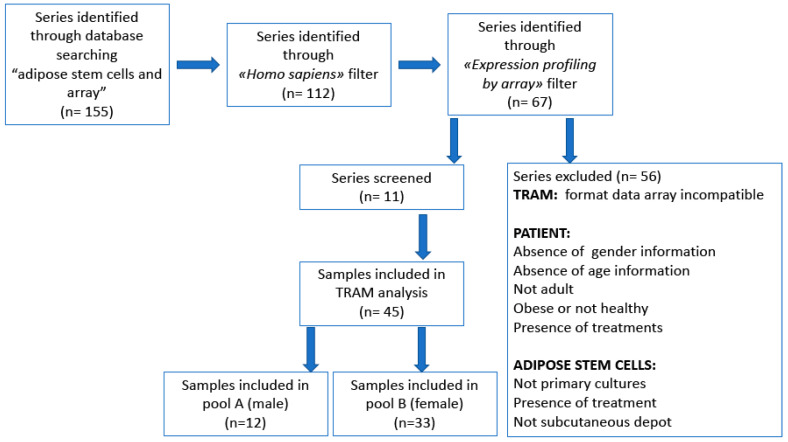
Flow diagram of data searching and selection strategy for Transcriptome Mapper (TRAM) meta-analysis.

Flow diagram of searching in GEO data repository and selection strategy for TRAM meta-analysis are resumed in Figure 1.

According to the criteria described in the “Materials and Methods” section, we included in TRAM meta-analysis a total of 11 series. The population analyzed in the “TRAM sex” analysis is composed of 12 male and 33 female hADSC samples with a donor age range of 31–71 and 18–69, respectively. On the other hand, the female samples analyzed in the “TRAM FGF” analysis are divided into 9 (cultured in a medium enriched by FGF) and 24 (cultured in a standard medium) hADSC female samples. The complete and detailed list of series and samples investigated in TRAM meta-analyses is shown in Table 1.

### 3.2. Default Map Mode Analysis in the “TRAM Sex”

TRAM analysis of pool A (12 male hADSC samples) vs. pool B (33 female hADSC samples) consisted, respectively of 211,979 and 992,099 data points (gene expression value), corresponding to 23,505 mapped loci (Supplementary Table S1). With the standard “Map” mode analysis, we obtained data about the differential expression of three segments. In particular, three chromosomes (Chr) showed segments with at least three over- or underexpressed protein-coding genes in hADSCs from different sex, as reported in Table 2.

The most expressed segment in male hADSCs is located in the short arm of Chr4 (4p12-p11) and includes three genes overexpressed in a statistically significant way (i.e., *q* < 0.05): *SLAIN2* (*SLAIN motif family member 2*; Gene ID: 57606), *SLC10A4* (*solute carrier family 10 member 4*; Gene ID: 201780) and *ZAR1* (*zygote arrest protein 1*; Gene ID: 326340), a gene with a known maternal effect.

In the Chr22 we can notice a segment with significant differential expression between hADSCs from donors of the opposite sex. In particular, three genes, mapping in 22q11.22, are overexpressed in male hADSCs: *IGLC1* (*immunoglobulin lambda constant 1*; Gene ID: 3537), *IGLJ3* (*immunoglobulin lambda joining 3;* Gene ID: 28831) and *BCR* (*breakpoint cluster region*; Gene ID: 613; one of the two genes forming the complex BCR-ABL, associated with the Philadelphia chromosome).

Moreover, in the long arm of the Chr7 (7q21.3), there is a distinct segment where three underexpressed genes in male hADSCs mapped: *TFPI2* (*tissue factor pathway inhibitor 2*; Gene ID: 7980), *GNGT1* (*G protein subunit γ transducin 1*; Gene ID: 2792) and *GNG11* (*G protein subunit γ 11*; Gene ID: 2791).

A systematic bibliographical search was performed for every significant over- or underexpressed gene listed in Table 2 to evaluate if any relationship between stem cells and sex/gender is known and/or if any other correlated information could be useful to better understand our obtained data. All the results are better presented and commented in the Discussion section.

### 3.3. Single Gene Level Map Mode in “TRAM Sex” and Gene Enrichment Analysis

“TRAM sex” analysis of pool A (male samples) and pool B (female samples) conducted with restricted parameters allowed to generate a list of loci that resulted differently expressed between the considered conditions (i.e., *q* < 0.05). In Table 3 we highlighted the twenty genes most over- and underexpressed in a statistical manner in male vs. female samples, considering those genes where the “data points” value was >5 (see Table S1).

The complete list of “TRAM sex” results obtained with the restricted setting is reported in Supplementary Table S2. Single gene level analysis of hADSC data generated a total of 8909 loci corresponding to 639 single transcripts with a significant altered expression.

At single gene level, the first 20 genes with the higher expression ratio in male samples are autosomal genes coding for proteins with diverse functions. The known gene *ITGB8* (*integrin subunit β 8*; Gene ID: 3696) mapping on Chr17 has the highest expression value (Table 3 and Table S2), followed by two other known genes, *ITGA8* (*integrin subunit α 8*; Gene ID: 8516) and *GALNT15 (polypeptide n-acetylgalactosaminyltransferase 15*; Gene ID: 117248), mapping, respectively on Chr10 and Chr3: interestingly, all these genes code for proteins involved in binding processes. Among the more underexpressed loci in male hADSCs in comparison with the female cells, there are genes coding for proteins with different functions and for one miRNA. The most underexpressed genes in male cells are *NPIPB3* (*nuclear pore complex interacting protein family member B3*; Gene ID: 23117; mapped on Chr16), member of the nuclear pore complex interacting protein family and *MT1HL1* (*metallothionein 1h like 1*; Gene ID: 645745; mapped on Chr1) encoding for a metallothionein. The most noticeable results shown in Table 3 are explained and reviewed in the Discussion section.

Finally, all the genes resulted as differentially expressed in male vs female hADSCs were analyzed for GO enrichment in ShinyGo v0.61 (Table S3). The pathways with the highest enrichment FDR in male hADSCs are related to cell adhesion and signaling, while in females the most significant activated processes are associated with cell metabolism and response to stimulus.

### 3.4. Default and Single Gene Level Map Mode Analyses in the “TRAM FGF”: Influence of FGF on Gene Expression of hADSCs

Since we chose, for the “TRAM sex” analysis, male and female hADSC samples cultured both with standard medium and with medium supplemented with FGF, we decided to perform an additional TRAM analysis (“TRAM FGF”) with the medium supplement as the only variable. We selected samples from pool B (only females) being the largest group of samples and therefore more reliable to obtain statistically solid data. We retain that “TRAM FGF” analysis could help us to better understand and reinforce “TRAM sex” results.

TRAM analysis of pool C (9 female hADSC samples cultured in FGF-supplemented medium) vs. pool D (24 female hADSC samples cultured in a standard medium without FGF) consisted, respectively of 152,220 and 839,878 data points (gene expression value), corresponding to 19,287 mapped loci (Supplementary Table S4). With the standard “Map” mode analysis, we obtained data about the differential expression of eight segments, belonging to six chromosomes, as reported in Table 4. Interestingly, the segments that refer to the chromosomes 19, 20 and 1 presented several significantly overexpressed RNA genes, belonging to the class of small nucleolar RNA (snoRNA). In some cases, the overexpression of a gene is associated with that of the paralogous genes. Some of these snoRNAs have been associated with specific diseases such as frontal sinusitis (*SNORD32A*—*small nucleolar RNA*, *C/D Box 32A*; Gene ID: 26819), mitochondrial myopathy (*SNORD35A*—*small nucleolar RNA*, *C/D Box 35A*; Gene ID: 26816) and laryngotracheitis (*SNORD76*—*small nucleolar RNA*, *C/D Box 76*; Gene ID: 692196) (information obtained by Gene Cards database).

In addition, two of the three segments mapping on chromosome 11 have at least three overexpressed genes and the other one three underexpressed genes. Even in this chromosome paralogous genes are co-regulated, as for example *OR8H2* (*olfactory receptor family 8 subfamily H member 2*; Gene ID: 390151) and *OR8H3* (*olfactory receptor family 8 subfamily H member 3*; Gene ID: 390152). This result indicates that the presence of FGF in hADSC culture medium can particularly influence the expression of genes located on this chromosome.

Noteworthy, the cytoband 4p12-p11 and in particular the genes *SLAIN2*, *SLC10A4* and *ZAR1* resulted significantly overexpressed in female hADSCs cultured in medium enriched with FGF compared to female hADSCs cultured in standard medium without FGF. These genes are also overexpressed in male hADSCs compared to female hADSCs, as reported in the 3.2 paragraph.

“TRAM FGF” analysis conducted with restricted parameters allowed to generate a list of loci that resulted differently expressed between the considered conditions (i.e., *q* < 0.05). The complete list is reported in Supplementary Table S5. Single gene level analysis of hADSC data generated a total of 8353 loci corresponding to 772 single transcripts with a significant altered expression.

**Figure 2:**
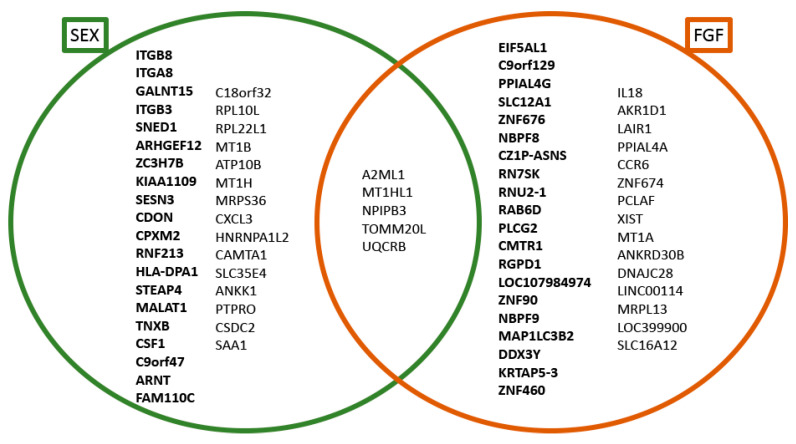
Venn diagram of the 40 most significantly regulated genes obtained with “TRAM sex” and “TRAM FGF” single gene level analysis. Genes in bold are those overexpressed in male (diagram “SEX”) or in cell supplemented with FGF (diagram “FGF”), while genes written not in bold are the underexpressed ones.

In Figure 2 we show the intersection between sex and FGF regulation, comparing the 20 most over-/underexpressed genes resulted from the two single gene level analyses (see, respectively Table 3 and Table S5). The five shared genes (*A2ML1*, *MT1HL1*, *NPIPB3*, *TOMM20L* and *UQCRB*) are not discussed as possible sex-specific loci.

## 4. Discussion

Sexual dimorphism affects several aspects of the biology of mammalians which evolved under adaptive and sexual pressure [1]. In humans, sex-biased differences are described at diverse structural hierarchical levels. Hip bone is structurally different in women as adaptation to birth [2], brain structure and functions vary between sexes [12] as well as the metabolism and immunological responses [18]. All these differences are not completely due to the levels of circulating hormones and could be ascribed also to the main difference between sexes, namely the karyotype. The male and female genomes are almost identical, except for the Y chromosome [26], but nevertheless sex-related differences arise at the molecular level due to the differential gene expression [27,28], alternative transcripts [29,30] and epigenetic modifications [31]. Under these points of view, all the cell types in a multicellular organism share the same genome and, at the same time, the identity of every cell type is determined by several molecular factors, including the genetic sex. The impact of SD at the cellular level is not completely understood, but literature indicates that sex could affect the properties of cells. For example, in a rat model, XY and XX neurons are differently susceptible to various cytotoxic agents in vitro and their programmed cell death proceeds differently, predominately through an apoptosis-inducing factor-dependent pathway in male neurons and via cytochrome c-dependent pathway in female cells [84]. Even the reaction to stress is different, with female cells more prone to undergo toward senescence pathways while the male counterpart expresses apoptotic markers [85]. The influence of sex on cell properties is particularly interesting in the field of stem cells and regenerative medicine, however, it is still poorly studied. By way of example, the cell sex of hAMSCs influences the uptake of nanoparticles, due to a different production of paracrine factors and the efficiency of cell reprogramming [57]. Noteworthy, the tendency to express differentiation/regeneration capabilities of adult stem cells is sex-related both in human and a murine model [52,55].

The aim of this work was to characterize the gene expression differences related to sex of hADSCs, considered a promising tool of regenerative medicine. In fact, hADSCs are multipotent stromal cells easily isolated from fat tissue during plastic surgery, they possess anti-inflammatory, immunomodulatory, immune tolerance and pro-angiogenic effects [58,59]. In order to perform a comparative transcriptomic analysis of male and female hADSC data, we used the TRAM (Transcriptome Mapper) software, a tool able to generate quantitative transcriptome maps, starting from publicly available microarray data from independent studies performed on different array platforms, through an intra- and inter-sample normalization (scaled quantile) method, useful to avoid possible batch effect [42,65]. In particular, TRAM analyses allowed us to identify several chromosomal segments and genes with significant differential expressions between two biologic conditions and the results were used to perform a systematic literature study.

The impact on cell transcriptome of several factors as the BMI status and/or the age of donors, the characteristics of the adipose tissue, as the depot or type of isolated fat and culture conditions, influenced the selection of microarray data to include in our “TRAM sex” analysis [86].

In particular, a recent study revealed that obesity causes changes in gene expression across several tissues, highlighting that BMI-related genes are not only adipose tissue specific [87]. Ronn and Colleagues showed the impact of both BMI and age on epigenetic modifications of genes candidate for metabolic diseases and cancer in human adipose tissue [88]. Moreover, the obese status, defined as BMI ≥ 30 kg/m^2^ [68,69], negatively affects the properties of ADSCs [89] and both murine and human BM-MSCs [90,91]. Obesity and its associated inflammation promote dysregulation of both adipocytes and hADSC plasticity [92].

Among the donor features that could affect hADSC dynamics, also age has been investigated by a plethora of studies, both in murine [93] and human models [94,95]. The heterogeneity of hADSC gene expression was also seen related to the type of adipose tissue used to obtain the stem cells, like the visceral or the subcutaneous adipose tissue [96,97] and also related to different cells of the same depot, excellently reviewed by Prieto-Gonzàles [95].

Lastly, culture conditions, as the medium and serum supplementation, can influence cell morphology, proliferation and gene expression [98,99,100]. For instance, FGF is usually used as MSC culture supplement due to its known effect on the preservation of their stemness properties as self-renewal and plasticity [101].

For all these reasons, we chose to consider data referred to ADSCs isolated only from subcutaneous fat (most of them referred to abdominal depot) of healthy, not-obese and adult subjects. In addition, where necessary, we have considered the putative influence of culture conditions, in particular of FGF addition.

### 4.1. Chromosomal Segments Overexpressed in Male hADSCs

The default “Map” mode analysis was conducted to identify chromosomal segments differently expressed between hADSCs derived from different sex donors. As reported in Table 2, the genomic regions with a sex-biased transcription refer to three chromosomes.

The most expressed regions in male hADSCs with respect to the female cells lie in the 4p12 and 22p11 genomic positions that show an expression ratio of 2.38 and 2.34, respectively (Table 2). Three genes mapping at 22p11 resulted significantly overexpressed in male hADSCs. Two of them code for immunoglobulins, *IGLC1* and *IGLJ3*. The third overexpressed gene is *BCR*. The cytoband 22p11 is well known to be involved in chromosomal abnormalities; *BCR* is directly involved in the reciprocal translocation with the *ABL1* gene on Chr9 that produces the Philadelphia chromosome and forms the fusion transcript *BCR-ABL1*, strictly associated with the chronic myeloid leukemia (CML) [102,103]. No information is obtained about *IGLC1* and/or *IGLJ3* expressions in stem cells, as well as for the *BCR* expression. Despite this, Nowak and coll. [104] in a SNP array analysis of tyrosine kinase inhibitor-resistant CML identified some secondary genomic abnormalities, including newly acquired and recurrent deletions of the IGLC1 locus. In addition, these genes have not yet been reported in research literature as being associated with a sex-biased expression and further investigations are required to validate these putative dimorphic expression patterns.

Three statistically significant overexpressed genes in male hADSCs were found to map in the cytoband 4p12-p11: *SLAIN2* and *SLC10A4*, coding for a protein important for microtubule (MT) dynamics and organization, and for a bile acid transporter and synaptic vesicle protein [105], respectively. No data are available connecting the overexpression with stem cell function or properties. Despite this, SLAIN2 strongly stimulated processive MT polymerization in interphase cells [106] and, in a murine cancer model, it is essential for mesenchymal cell invasion in 3D culture [107] while, in rats, *SLC10A4* is expressed by cholinergic neurons even if it is not reported as choline transporter [108]. The third gene that lies in the cytoband 4p12-p11 is *ZAR1*, a gene known for its maternal effect and responsible for the zygote–embryo transition and indicated as tumor suppressor in cancer cell lines by inhibiting the cell cycle progression [109]. It is described in literature its female sex-biased expression both in *Scatophagus argus* and eels [110,111]. Interestingly, *ZAR1* resulted to be overexpressed in hADSCs derived from male donors in comparison with the female ones. Its transcription is found to occur at a low level in the reference dataset and, despite its known maternal effect, *ZAR1* shows a peak of expression in testis tissue [112]

### 4.2. Chromosomal Segments Overexpressed in Female hADSCs Cultured in Medium Enriched with FGF

In the “TRAM sex” analysis, nine of the total 33 female samples (see Table 1) and nine of the total 12 male samples (data not shown) derived from hADSCs cultured with FGF. Thus, in both sex and FGF TRAM analyses *SLAIN2*, *SLC10A4* and *ZAR1* genes are overexpressed in the pool where more FGF culture supplement was present. We could, therefore, speculate that these differences in gene expression, should be most probably influenced by FGF culture supplement, even if a male “TRAM FGF” analysis could help us in better understanding (to date, the number of available male samples is not sufficient to provide statistically reliable data with TRAM software). *SLAIN2* and *SLC10A4* encode for a protein important for MT dynamics and organization and for a bile acid transporter and synaptic vesicle protein [105], respectively. No data are available connecting the overexpression with stem cell function or properties. Despite this, SLAIN2 strongly stimulated processive MT polymerization in interphase cells [106] and, in a murine cancer model, it is essential for mesenchymal cell invasion in 3D culture [107] while, in rats, *SLC10A4* is expressed by cholinergic neurons even if it is not reported as choline transporter [108]. The third gene that lies in the cytoband 4p12-p11 is *ZAR1*, a gene known for its maternal effect and responsible for the zygote–embryo transition and indicated as tumor suppressor in cancer cell lines by inhibiting the cell cycle progression [109]. It is described in literature its female sex-biased expression both in *Scatophagus argus* and eels [110,111], but also its a peak of expression in testis tissue [112]. Details and further discussion of “TRAM FGF” results will be the subject for our future investigations.

### 4.3. Chromosomal Segments Underexpressed in Male hADSCs

One chromosomal segment is underexpressed in hADSCs derived from male patients with respect to the female counterpart. In fact, in the cytoband 7q21.3 there are three male underexpressed genes: *TFPI2* that encodes for a member of the Kunitz-type serine proteinase inhibitor family, *GNGT1* that encodes the γ subunit of transducin, a guanine nucleotide-binding protein and *GNG11* that is a member of the heterotrimeric G protein complex and plays a role in this transmembrane signaling system.

*TFPI2*, as a suppressor gene, is known to be dysregulated in multiple human disorders, including preeclampsia [113,114] and various cancers, where its expression is inversely related to an increasing grade of malignancy [115,116]. In gastric cancer cell lines, it was found to be one of the most densely methylated genes with a negative correlation with the survival of patients [117]. In the same work, *TFPI2* expression was found to be associated with sex, in particular it was more expressed in males; unfortunately, we were not able to in-depth speculate about the different trend found in our work because of the different cellular model. In two more studies about the role of TFPI2 in tumors (bladder cancer and renal cell carcinoma), a pro-apoptotic role was demonstrated [115,118]. Moreover, *TFPI2* overexpression was found to strongly inhibit the proliferation and migration of vascular smooth muscle cells [116] and to reduce endothelial cell proliferation induced by vascular endothelial growth factor [119]. Although this information derives from different experimental models, we could infer that *TFPI2* plays, also in male and female hADSCs, a role in apoptosis, proliferation and migration. The difference in its expression level brings to a distinct behavior between male and female cells in the cell cycle regulation as well as in the migration, two key processes of stem cells, as the differentiation process. In particular, following this information, female hADSCs should result to be more prone to undergo in apoptosis and to be less inclined to proliferate and migrate.

*GNG11* and *GNGT1* are two paralog genes, both encoding for a specific G protein subunit γ. In literature, the influence of *GNG11* and *GNGT1* genes on stem cell biology or the relation to the genetic sex is poorly investigated. About *GNGT1* and sex, it was found to be overexpressed in brains of female rare minnows [120], as a component of the phototransduction pathway, underlining its possible role in the brain sexually dimorphism. These data confirm our result even if in another cell type and organism, however without giving us information about its putative role in male and female hADSCs. In a recent study, GNG11 resulted as the best candidate protein to have an inhibitory effect on the cervical cancer [121], underlining the important role in a female organ. Moreover, GNG11 is known to induce cellular senescence in normal human diploid fibroblasts and to suppress cell growth with the induction of reactive oxygen species and abnormal nuclear morphology in SUSM-1 cells [122]. Therefore, its downregulation in male hADSCs could suggest a possible different mechanism of cell cycle regulation between MSCs of different sex, leading to hypothesize that female hADSCs are less inclined to proliferate, as it is also suggested by the coherent differential expression of *TFPI2*.

### 4.4. Genes Differentially Expressed in Male and Female hADSCs

By reducing the sliding window shift of “TRAM sex” analysis we were also able to identify loci, each corresponding to a single gene (TRAM segment window of 12,500 bp), that are differently expressed in male and female hADSCs. In Table 3 we listed the 20 genes most over- and underexpressed in male hADSCs with respect to the female counterpart.

We decided to focus our discussion on the seven most overexpressed genes in male hADSCs in comparison with female cells. Interestingly, in the list of the top 20 male overexpressed transcripts in male vs. female cells there are no Y-linked genes, as commonly expected in a sex-specific analysis and already reported in another TRAM conducted on male and female *substantia nigra* cells [42].

For these seven overexpressed genes, a literature screening was performed as described in the “Materials and Methods” section. Three integrins, *ITGB8*, *ITGA8* and *ITGB3* (*integrin subunit β 3*; Gene ID: 3690) resulted to be among the most significantly overexpressed genes in male samples. These genes encode for integrin subunits, whose function is the cell–cell and cell-extracellular matrix (ECM) interaction. Roles of integrins in stem cells biology are known. By way of example, *ITGB8* plays a crucial role in chondrogenesis of hMSCs [123] and its underexpression is reported during the adipogenic differentiation of MSCs [124]. The evidence of *ITGB8* overexpression in male hADSC samples could suggest a predisposition to the chondrogenic commitment of male MSCs rather than the adipogenic one. This hypothesis is coherent with the detection of *CXCL3* underexpression (discussed below).

The gene *ITGA8*, among other studies, was studied in multiple myeloma cell lines since it was discovered its high expression in patients with early relapse [125]. The *ITGA8* overexpression in this cell model was associated with an induction of stemness features and epithelial-mesenchymal transition-related phenotypes. These, consequently, enhanced migration and invasion abilities, which are crucial to multiple myeloma pathogenesis; this evidence could be transposed into the hADSC model where ITGA8 could be seen as a factor promoting stemness. If so, hADSCs derived from male donors would have more stemness characteristics.

Concerning *ITGB3*, it is a positive surface marker of hemogenic ECs both in hESCs and mouse embryo during hematopoiesis [126] and its expression together with *itga2b* gradually increases in differentiating mouse hematopoietic stem cells [127]. Noteworthy, it is reported that *ITGB3* is overexpressed in the porcine endometrium supplying female fetuses [128]. The fact that in the analyzed hADSC samples three integrins are differently expressed between cells of diverse sex suggests that interactions between cells and their microenvironment, as well as the mechano-transductions and communication of stem cells, could be a sex-related influence.

Another overexpressed gene in male vs. female hADSCs is *GALNT15.* The literature search did not give any interesting results about this specific gene, but we found a recent study [129] aimed to screen several differentially expressed genes for two types of MSC differentiation, as osteoblastic and adipocytic differentiation, regarding other members of the *GALNT* gene group. In particular, *GALNT1* was found to be upregulated in adipocytic differentiation, leading to hypothesize that it could have a possible role in adipogenesis. We therefore could speculate about a possible role also of GALNT15 in hADSC adipogenesis; if it would be confirmed in a proper research, it could reinforce the idea that adipogenesis is differently regulated in male and female.

*SNED1* (*sushi, nidogen and EGF like domains 1*; Gene ID: 25992) also known as *IRE-BP1* (*insulin-responsive sequence DNA-binding protein 1*), activates insulin-responsive genes *IGF-I*, *IGFBP-1* and *IGFBP-335* and consequently it is expressed in insulin-responsive tissues such as fat and muscle [130]. Very little literature was found about it. In a wide study aimed to investigate the association between measures of body size and body composition with DNA methylation on a genome-wide scale in 374 preschool children, methylated probes located in *SNED1* was found to be significantly inversely associated with BMI, fat mass and fat mass index [131]. We could, therefore, assume that SNED1 plays a sex-influenced role in hADSCs associated with BMI and fat mass.

*ARHGEF12* (*Rho guanine nucleotide exchange factor 12*; Gene ID: 23365) is a Rho GTPases and is overexpressed in male derived hADSCs compared to the females. The encoded protein may form a complex with G protein and mediate several cellular processes, suggesting that G protein signaling may be fashioned within the context of a cell-sex influence. Recently, it has been shown bioinformatically that *ARHGEF12* is part of a network of genes and miRNAs that regulates axon regeneration [132] while in zebrafish the knockout of *Arhgef12* resulted in a defective erythropoiesis [133]. No correlations between *ARHGEF12* and sex are reported in literature until now.

Another gene overexpressed in male, encoding for a zinc-finger protein, is *ZC3H7B* (*zinc finger CCCH-type containing 7B*; Gene ID: 23264), a gene involved in miRNA biogenesis [134]. Zinc finger domain confers to the proteins the capacity to bind with nucleic acids and it is conceivable that cell sex could affect this type of interaction in hADSCs.

TRAM analysis at single gene level also allowed us to identify underexpressed genes in males compared to females. As for the most overexpressed genes in male hADSCs in comparison with female cells, we decided to perform a systematic bibliographical search for the seven most underexpressed genes in male cells (also listed in Table 3). Anyway, very few information about these genes were linked to stem cells and/or to sex. Interestingly, it has been reported that in human HT29 cells after the administration of malignant free cell DNA the metallothionein *MT1HL1* (*metallothionein 1H like 1*; Gene ID: 645745, also known as *MT1P2*), resulted overexpressed together with *MALAT1* (*metastasis associated lung adenocarcinoma transcript 1*; Gene ID: 378938), a gene overexpressed in male hADSCs (Table 3) [135]. Moreover, the bibliographical search about *A2ML* (*α-2-macroglobulin like 1*; Gene ID: 144568) and *RPL10L* (*ribosomal protein L10 like*; Gene ID: 140801) highlighted some connections to sex. In a study of Burgener and Colleagues, *A2ML* resulted over-abundant in HIV-1-resistant women cervicovaginal lavage fluid (but no information are available from male partners) [136], while it has been demonstrated that the expression of *RPL10L* is restricted to the testis both in mice and humans, and it is required to compensate for *Rpl10* silencing resulting from meiotic sex chromosome inactivation in a mouse model [137].

Moreover, the overall analysis of the underexpressed genes in male reported in Table 3, allowed us to understand that some of them play important roles in inflammation, in the stem cell differentiation as for example the adipogenic, neurogenic and myogenic commitment and in stem cell adhesion (Table 3).

*CXCL3* (*C-X-C motif chemokine ligand 3*; Gene ID: 2921) encodes for a secreted growth factor that plays an important role in inflammation—and as a chemoattractant for neutrophils—it directly participates to the immunosuppressive properties of MSCs, by inhibiting proliferation and increasing apoptosis in T cells [136]. The underexpression of *CXCL3* and the overexpression of immunoglobulin genes (i.e., *IGLC1* and *IGLJ3*) in male hADSCs, corroborate the hypothesis that inflammatory and immunosuppressive properties are sex-dependent in hADSCs.

In addition, *CXCL3,* as other chemokines, is implicated in cell differentiation. Research by Kusuyama et al. (2016) has demonstrated that *CXCL3* was the most highly expressed gene in mature adipocytes. Moreover, the addition of CXCL3 to mouse preadipocyte cell line 3 T3-L1 induced cells to differentiate, also significantly promoting the increase of crucial adipogenic markers. Conversely, gene knockdown of *CXCL3* inhibited the course of adipogenic differentiation. Taken together, these studies indicated CXCL3 as an adipokine that facilitates adipogenesis [137]. Considering also other TRAM results, we could speculate that adipogenesis, promoted by CXCL3, is more favorite in females.

*ANKK1* (*ankyrin repeat and kinase domain containing 1*; Gene ID: 255239) encodes for a protein that belongs to the Ser/Thr protein kinase family and to the protein kinase superfamily that is involved in signal transduction pathways. Its relation with neuropsychiatric disorders, its localization in neural progenitors and its correlation with the cell cycle, suggested that ANKK1 could participate in neural development [138] and in the regulation of the metabolism of muscles during development and in adulthood [139]. Even *CSDC2* (*cold shock domain-containing C2*; Gene ID: 27254) that encodes for an RNA-binding protein is involved in the myogenic differentiation. Meyer and collaborators identified in murine skeletal myoblasts PMI28 an inverse regulation of Csdc2 expression during in vitro skeletal myoblast differentiation [140].

Many studies have then demonstrated the role of *CAMTA1* (*calmodulin binding transcription activator 1*; Gene ID: 23261) in inhibiting proliferation and inducing differentiation. By way of example, research by Borer and Colleagues showed that the cell–cell communications between MSCs and the adjacent co-cultured neonatal cardiomyocytes, induced Ca(2+) signals that activated a myocardial gene program in the stem cells via an early Ca(2+)-dependent intermediate upregulation of CAMTA1 [141]. Moreover, CAMTA1 was studied in neuroblastoma cells, where it slowed cell proliferation and induced neurite-like processes by activating the markers of neuronal differentiation [142].

The gene *PTPRO* (*protein tyrosine phosphatase receptor type O*; Gene ID: 5800) that encodes for a developmentally regulated protein, also detected in human CD34+ bone marrow cells, is involved in megakaryocytopoiesis [143] and has a putative role in stem cell adhesion [144], suggesting together with other genes retrieved in “TRAM sex” analysis (as *integrins* and *TFPI2*) that adhesion and motility could be sex-related in hADSCs.

It is important to underline that our study of the over- and underexpressed transcripts in male vs. female cells confirm the GO enrichment analysis (see Table S3) where the pathways with the highest enrichment FDR in male hADSCs are related to cell adhesion and signaling. In fact, these are fundamental pathways for the cell communication and commitment, processes that have shown a different involvement in males compared to females.

Finally, it is necessary to highlight that some of the most male underexpressed genes reported in Table 3, like *UQCRB*, *TOMM20L*, *A2ML1*, *MT1HL1* and *NPIPB3,* resulted probably influenced from FGF supplementation (see Table S5 and Figure 2). Therefore, further investigations are needed to understand if also sex plays a role in their expression.

## 5. Conclusions

An in-depth study of the main TRAM results allowed us to identify chromosomic segments and genes that are differentially expressed in hADSCs derived from male and female donors and to identify putative hADSC sex-related properties.

Taken together, the TRAM meta-analysis results lead us to hypothesize that the donor sex of hADSCs is an important variable influencing several biologic processes, as inflammation, cell adhesion and senescence and stem cell properties, as migration, proliferation and immunomodulation and differentiation.

Differential immunomodulation capacity could be extrapolated from the evidence that some inflammation-related genes, including some C-X-C motif ligands and immunoglobulin genes (as *IGLC1* and *IGLJ3*), are, respectively, under- and overexpressed in male derived hADSCs, compared to the female ones.

The underexpression of *CXCL3* suggests an inference of male cell differentiative potency, as well as those of *ANKK1*, *CAMTA1* and *CSDC2* involved in different stem cell commitment. In fact, in the last decade, chemokines have been implicated in cell differentiation [145,146] and *CXCL3* positively regulates adipogenic differentiation in mouse preadipocyte and MSC cell lines [139]. This evidence together with TRAM results on *CXCL3* underexpression suggests that human male derived ADSCs could be less prone to differentiate toward the adipogenic fate than the female counterpart.

Among the most over- or underexpressed genes obtained from TRAM analysis (as the three integrins and *PTPRO* gene), there are some genes involved in the cell-cell or cell-ECM adhesion process, which suggest possible different ways of communication between male and female hADSCs.

Finally, many genes involved in the mechanism of cell cycle regulation (as *TFPI2*, *GNG11*, *ANKK1* and *CAMTA1*) denote that stem cell proliferation and migration is probably a sex-related process in MSCs.

These in silico results, if confirmed with wet-lab experiments, could be exploited for the implementation of tools for the use of hADSCs in regenerative medicine in order to optimize the cell therapy practice.

## Figures and Tables

**Table 1:** Main features of the samples selected for the meta-analyses with TRAM software.

GEO Series	GEO Platform ID	Sex	Samples	Age	Regional Depot	Pool	PMID
GSE8954	GPL201	M	GSM226880	40.2 ± 14.2	Abdomen	A	20849362 [76]
		M	GSM226881	40.2 ± 14.2		A	
		F	GSM226882	40.2 ± 14.2		B (D)	
		F	GSM226883	40.2 ± 14.2		B (D)	
GSE12399	GPL201	M	GSM311246	52	Abdomen	A	N/A
		F	GSM311250	42		B (C)	
GSE18201	GPL201	F	GSM455150	42	Abdomen	B (C)	20605541 [77]
		M	GSM455151	49		A	
		F	GSM455152	51		B (C)	
GSE19773	GPL7363	F	GSM493884	(18–39)	Abdomen	B (D)	20410135 [78]
		F	GSM493885	(18–39)		B (D)	
GSE24433	GPL571	F	GSM602158	Adult	Abdomen	B (D)	22669576 [79]
GSE37324	GPL6244	F	GSM916113	69	Abdomen	B (C)	23526958 [80]
		F	GSM916114	69		B (C)	
		F	GSM916116	69		B (C)	
		M	GSM916117	71		A	
		M	GSM916119	71		A	
		M	GSM916123	71		A	
		F	GSM916124	62		B (C)	
		F	GSM916128	62		B (C)	
		M	GSM916129	53		A	
		F	GSM916130	62		B (C)	
		M	GSM916132	56		A	
		M	GSM916135	53		A	
		M	GSM916136	53		A	
GSE48220	GPL6480	F	GSM1173058	39	N/A*	B (D)	N/A
		F	GSM1173059	31		B (D)	
		F	GSM1173060	35		B (D)	
		F	GSM1173061	32		B (D)	
		F	GSM1173062	29		B (D)	
		F	GSM1173063	37		B (D)	
GSE48774	GPL6884	F	GSM1184376	(24–40)	Thigh/buttock	B (D)	26462465 [81]
		F	GSM1184377	(24–40)		B (D)	
		F	GSM1184378	(24–40)		B (D)	
GSE57538	GPL13158	F	GSM1384338	(36–45)	Abdomen	B (D)	25437437 [82]
		F	GSM1384339	(36–45)		B (D)	
		F	GSM1384340	(36–45)		B (D)	
GSE77272	GPL10558	M	GSM2047165	31	Abdomen	A	27224250 [83]
		F	GSM2047164	56		B (D)	
		F	GSM2047166	19		B (D)	
		F	GSM2047167	57		B (D)	
GSE98421	GPL570	F	GSM2595136	30	Abdomen	B (D)	N/A
		F	GSM2595137	19		B (D)	
		F	GSM2595138	29		B (D)	
		F	GSM2595139	33		B (D)	

Gene Expression Omnibus (GEO) series and platform ID—ID numbers as stated in the GEO database; Sex—male (M) and female (F) hADSC donor samples; Samples—sample named as stated in the GEO database; Age—donor age declared in GEO series or in related manuscript as the specific/range age or as the mean value of all the indicated donors’ ages and standard deviation (SD); Adult—subject >18 years old; Pool—in order to perform the described analyses all samples were divided into pools (see “Materials and Methods” Section; “TRAM analysis” paragraph); PMID—PubMed identifier number of the reference reported in the GEO database; N/A—information not available; *—female liposuction.

**Table 2:** Chromosomal segments statistically significant in TRAM “Map” mode analysis of male vs. female hMSCs derived from adipose tissue (hADSCs) samples.

Chr	Location	Segment Start	Segment End	Expression Ratio	*q*-Value	Genes in the Segment
Chr 4	4p12-p11	48,250,001	48,750,000	2.38	1.73 × 10^−4^	*TEC*− ***SLAIN2***+ ***SLC10A4***+ ***ZAR1***+ *FRYL*+
Chr22	22q11.22	22,750,001	23,250,000	2.34	1.70 × 10^−4^	***IGLC1***+ ***IGLJ3***+ *RSPH14*+ *GNAZ*+ *RAB36*+ ***BCR***+
Chr7	7q21.3	93,500,001	94,000,000	0.50	8.67 × 10^−5^	*CALCR*− ***TFPI2***− ***GNGT1***− ***GNG11***− *BET*−

Statistically significant segments obtained with TRAM “Map” mode analysis (default parameters) by comparing pool A (male derived hADSCs) vs. pool B (female derived hADSCs). The resulted segments are sorted by decreasing the expression ratio. Chr—chromosome; Location—segment cytoband derived from the first mapped gene within the segment; segment start/end—chromosomal coordinates for each segment; Genes in the segment—bold and +—overexpressed gene; bold and −—underexpressed gene; ‘+’ or ‘−’—gene expression value higher or lower than the median value; respectively.

**Table 3:** List of the twenty genes significantly most over- or underexpressed (all significantly, with *q* < 0.05) in male vs. female hADSC samples.

Gene Name	Gene ID	Function (GO Terms)	Chr	Location	Segment Start	Segment End	Expression Ratio
*ITGB8*	3696	extracellular matrix protein binding	Chr7	7p21.1	20,318,751	20,425,000	8.88
*ITGA8*	8561	metal ion binding	Chr10	10p13	15,506,251	15,731,250	6.89
*GALNT15*	117248	carbohydrate binding	Chr3	3p25.1	16,162,501	16,256,250	5.93
*ITGB3*	3690	extracellular matrix protein binding	Chr17	17q21.32	47,243,751	47,256,250	5.97
*SNED1*	25992	Notch binding	Chr2	2q37.3	240,987,501	241,068,750	5.68
*ARHGEF12*	23365	G protein-coupled receptor binding	Chr11	11q23.3	120,337,501	120,500,000	5.51
*ZC3H7B*	23264	RNA binding	Chr22	22q13.2	41,306,251	41,362,500	5.42
*KIAA1109*	84162	protein binding	Chr4	4q27	122,143,751	122,375,000	5.27
*SESN3*	143686	protein binding	Chr11	11q21	95,231,251	95,243,750	5.23
*CDON*	50937	protein binding	Chr11	11q24.2	125,950,001	126,075,000	5.04
*CPXM2*	119587	zinc ion binding	Chr10	10q26.13	123,737,501	123,956,250	4.97
*RNF213*	57674	ATPase activity	Chr17	17q25.3	80,256,251	80,350,000	4.96
*HLA-DPA1*	3113	peptide antigen binding	Chr6	6p21.32	33,056,251	33,075,000	4.89
*STEAP4*	79689	FAD binding	Chr7	7q21.12	88,268,751	88,318,750	4.79
*MALAT1*	378938	*NA*	Chr11	11q13.1	65,487,501	65,512,500	4.72
*TNXB*	7148	collagen fibril binding	Chr6	6p21.33-p21.32	32,043,751	32,106,250	4.68
*CSF1*	1435	cytokine activity	Chr1	1p13.3	109,900,001	109,937,500	4.64
*C9orf47*	286223	*NA*	Chr9	9q22.1	88,981,251	89,000,000	4.55
*ARNT*	405	DNA-binding transcription factor activity	Chr1	1q21.3	150,812,501	150,887,500	4.49
*FAM110C*	642273	α-tubulin binding	Chr2	2p25.3	31,251	56,250	4.46
*SAA1*	27254	transcription factor binding	Chr11	11p15.1	18,256,251	18,268,750	0.14
*CSDC2*	27254	transcription factor binding	Chr22	22q13.2	41,550,001	41,575,000	0.14
*PTPRO*	5800	protein binding	Chr12	12p13-p12	15,593,751	15,606,250	0.14
*ANKK1*	255239	ATP binding	Chr11	11q23.2	113,387,501	113,406,250	0.13
*SLC35E4*	339665	antiporter activity	Chr22	22q12.2	30,631,251	30,681,250	0.12
*CAMTA1*	23261	sequence-specific DNA binding	Chr1	1p36.31-p36.23	6,775,001	7,768,750	0.11
*HNRNPA1L2*	144983	RNA binding	Chr13	13q14.3	52,606,251	52,650,000	0.11
*CXCL3*	2921	chemokine activity	Chr1	1q32.1	74,025,001	206,900,000	0.11
*MRPS36*	92259	structural constituent of ribosome	Chr5	5q13.2	69,212,501	69,231,250	0.10
*MT1H*	4496	metal ion binding	Chr16	16q13	56,668,751	56,681,250	0.10
*ATP10B*	23120	ATP binding	Chr5	5q34	160,556,251	160,937,500	0.10
*MT1B*	4490	metal ion binding	Chr16	16q13	56,643,751	56,656,250	0.07
*RPL22L1*	200916	RNA binding	Chr3	3q26.2	170,856,251	170,881,250	0.08
*RPL10L*	140801	structural constituent of ribosome	Chr14	14q21.2	46,643,751	46,662,500	0.08
*C18orf32*	497661	protein binding	Chr18	18q21.1	49,468,751	49,487,500	0.07
*UQCRB*	7381	protein binding	Chr8	8q22.1	96,218,751	96,237,500	0.06
*TOMM20L*	387990	mitochondrion targeting sequence binding	Chr14	14q23.1	58,387,501	58,406,250	0.06
*A2ML1*	144568	endopeptidase inhibitor activity	Chr12	12p13.31	8,812,501	8,893,750	0.06
*MT1HL1*	645745	metal ion binding	Chr1	1q43	236,993,751	237,012,500	0.05
*NPIPB3*	23117	molecular function	Chr16	16p12.2	21,393,751	21,437,500	0.02

TRAM “Map” mode analysis (single gene level parameters) comparing pool A (male derived hADSCs) vs. pool B (female derived hADSCs). The list is referred to the twenty most over- and underexpressed genes resulted with a segment window of 12,500 bp, considering genome median analysis (see full results in Supplementary Information section). Genes are sorted by decreasing the expression ratio. Gene ID—gene identification number in gene NCBHI; Chr—chromosome; Function—gene ontology function; N/A—information not available; location—segment cytoband derived from the first mapped gene within the segment; segment start/end—chromosomal coordinates for each segment.

**Table 4:** Chromosomal segments statistically significant in TRAM “Map” mode analysis of hADSC female samples with fibroblast growth factor (FGF) vs. hADSC female samples without FGF.

Chr	Location	Segment Start	Segment End	Expression Ratio	*q*-Value	Genes in the Segment
Chr11	11p15.5	1,250,001	1,750,000	4.44	6.13 × 10^−4^	*MUC5B*+ *TOLLIP*+ *BRSK2*+ *MOB2*− *DUSP8*+ *KRTAP5-AS1*+ ***KRTAP5-1***+ ***KRTAP5-2***+ ***KRTAP5-3***+ *KRTAP5-5*+ *FAM99A*− *KRTAP5-6*+ ***IFITM10***+
Chr19	19q13.33	49,250,001	49,750,000	4.44	8.91 × 10^−3^	*SLC6A16*+ *Hs.660609*+ *CD37*+ *TEAD2*+ *DKKL1*− *CCDC155*+ ***PTH2***+ *SLC17A7*− *PIH1D1*− *ALDH16A1*− *FLT3LG*+ *RPL13A*− ***SNORD32A***+ ***SNORD33***+ *SNORD34*+ ***SNORD35A***+ *RPS11*− *FCGRT*− *RCN3*+ *NOSIP*− *PRRG2*− *PRR12*+ *RRAS*− *SCAF1*− *IRF3*+ *BCL2L12*− *PRMT1*− *CPT1C*− *TSKS*+
Chr20	20q11.23	38,000,001	38,500,000	4.23	1.26 × 10^−5^	*TTI1*+ ***RPRD1B***+ ***TGM2***+ *KIAA1755*+ *BPI*− *LBP*+ *SNHG17*− *SNORA71B*+ *SNORA71A*+ ***SNORA71C***+ ***SNORA71D***+ *SNHG11*− *SNORA60*+ ***RALGAPB***+
Chr1	1q25.1	173,750,001	174,250,000	4.20	7.52 × 10^−3^	*KLHL20*+ *CENPL*− *DARS2*+ *SNORD47*+ *SNORD80*+ *SNORD79*+ ***SNORD78***+ *SNORD44*+ *SNORD77*− ***SNORD76***+ ***SNORD74***+ *ZBTB37*+ *SERPINC1*− *RC3H1*+ *RABGAP1L*+
Chr11	11p15.5	1,500,001	2,000,000	4.17	1.10 × 10^−3^	*DUSP8*+ *KRTAP5-AS1*+ ***KRTAP5-1***+ ***KRTAP5-2***+ ***KRTAP5-3***+ *KRTAP5-5*+ *FAM99A*− *KRTAP5-6*+ ***IFITM10***+ *CTSD*+ *SYT8*+ *TNNI2*+ *LSP1*− *TNNT3*+ *MRPL23*− *H19*+
Chr4	4p12-p11	48,250,001	48,750,000	3.95	5.97 × 10^−4^	*TEC*− ***SLAIN2***+ ***SLC10A4***+ ***ZAR1***+ *FRYL*+
Chr11	11q12.1	56,000,001	56,500,000	0.39	1.10 × 10^−3^	*OR5AS1*− *OR8I2*− ***OR8H2***− ***OR8H3***− *OR8J3*− *OR8K5*− *OR5J2*− *OR5T2*− ***OR5T3***− ***OR5T1***− *OR8H1*− *OR8K3*− *OR8K1*− *OR8J1*− *OR8U1*− *OR5R1*− *OR5M9*− *OR5M3*− *OR5M8*−
Chr7	7p21.1	16,500,001	17,000,000	0.36	2.98 × 10^−4^	*ANKMY2*− ***BZW2***− *TSPAN13*− ***AGR2***− ***AGR3***−

Chromosomal segments resulted by “TRAM FGF” “Map” mode analysis (default parameters) by comparing pool C (female hADSCs cultured in a medium enriched by FGF) vs. pool D (female hADSCs cultured in standard medium). The resulted segments are sorted by decreasing the expression ratio. chr—chromosome; location—segment cytoband derived from the first mapped gene within the segment; segment start/end—chromosomal coordinates for each segment; genes in the segment—bold and +—overexpressed gene; bold and −—underexpressed gene; ‘+’ or ‘−’—gene expression value higher or lower than the median value, respectively.

## Supplementary Materials

The following are available online at https://www.mdpi.com/2073-4425/11/8/909/s1, Table S1: List of 23,505 TRAM mapped loci for which an expression value A/B was calculated (hADSC samples from male vs. female). Loci are sorted in descending order. Gene name: official gene symbol as indicated in gene database; Chr: chromosome; data points: number of spots associated with an expression value for the locus; expression A or B: gene expression mean value of all data available for a locus; expression A/B: gene expression ratio of value A/value B; SD: standard deviation of the expression value indicated as percentage of the mean; Table S2: Map mode analysis at single gene level of pool A (hADSCs from male subjects) vs. pool B (hADSCs from female subjects). The 8909 resulting loci are sorted in descending order of expression ratio (A/B). Gene name: official gene symbol as indicated in gene database (the genes in bold are the over- or underexpressed in a statistical manner); Chr: chromosome; location: segment cytoband; segment start/end: chromosomal coordinates for each segment; expression A/B: gene expression ratio as mean value of all data available for a locus in pool A on pool B; q: *p*-value corrected for FDR (false discovery rate) of the segment; Table S3: ShinyGo enrichment analysis of genes differentially expressed in hASC male vs female. Gene ontology (GO) functional analysis of differentially expressed genes resulted from TRAM analysis at a single gene level. The GO enrichment analysis is based on Biologic Process functional categories of the ShinyGo v0.61 software. The results, ranked by FDR (false discovery rate) in the table, were obtained with a *p*-value cutoff (FDR) = 0.05 and 20 as number of most significant terms to show; Table S4: List of 19,287 TRAM mapped loci for which an expression value C/D was calculated (hADSC female +FGF vs. hADSC female -FGF). Loci are sorted in descending order. Gene name: official gene symbol as indicated in gene database; Chr: chromosome; data points: number of spots associated with an expression value for the locus; expression C or D: gene expression mean value of all data available for a locus; expression C/D: gene expression ratio of value C/value D; SD: standard deviation of the expression value indicated as percentage of the mean; Table S5: Map mode analysis at single gene level of pool C (hADSC female +FGF) vs. pool D (hADSC female -FGF). The 8353 resulting loci are sorted in descending order of expression ratio (C/D). Gene name: official gene symbol as indicated in gene database (the genes in bold are the over- or underexpressed in a statistical manner); Chr: chromosome; location: segment cytoband; segment start/end: chromosomal coordinates for each segment; expression C/D: gene expression ratio as mean value of all data available for a locus in pool C on pool D; q: *p*-value corrected for FDR (false discovery rate) of the segment.
